# Three-Dimensional Reconstruction and Visualization of Underwater Bridge Piers Using Sonar Imaging

**DOI:** 10.3390/s24144732

**Published:** 2024-07-21

**Authors:** Jianbin Luo, Shaofei Jiang, Yamian Zeng, Changqin Lai

**Affiliations:** 1 College of Civil Engineering, Fuzhou University, Fuzhou 350108, China; ljb312@fzu.edu.cn (J.L.);; 2 College of Civil Engineering, Quanzhou University of Information Engineering, Quanzhou 362008, China

**Keywords:** underwater bridge pier, sonar imaging, three-dimensional reconstruction

## Abstract

The quality of underwater bridge piers significantly impacts bridge safety and long-term usability. To address limitations in conventional inspection methods, this paper presents a sonar-based technique for the three-dimensional (3D) reconstruction and visualization of underwater bridge piers. Advanced MS1000 scanning sonar is employed to detect and image bridge piers. Automated image preprocessing, including filtering, denoising, binarization, filling, and morphological operations, introduces an enhanced wavelet denoising method to accurately extract the foundation contour coordinates of bridge piers from sonar images. Using these coordinates, along with undamaged pier dimensions and sonar distances, a model-driven approach for a 3D pier reconstruction algorithm is developed. This algorithm leverages multiple sonar data points to reconstruct damaged piers through multiplication. The Visualization Toolkit (VTK) and surface contour methodology are utilized for 3D visualization, enabling interactive manipulation for enhanced observation and analysis. Experimental results indicate a relative error of 13.56% for the hole volume and 10.65% for the spalling volume, demonstrating accurate replication of bridge pier defect volumes by the reconstructed models. Experimental validation confirms the method’s accuracy and effectiveness in reconstructing underwater bridge piers in three dimensions, providing robust support for safety assessments and contributing significantly to bridge stability and long-term safety assurance.

## 1. Introduction

To ensure the safety and reliability of underwater bridge piers, regular physical examinations and monitoring are crucial. The harsh hydrogeological environment and long-term exposure to water erosion and human activities make these foundations susceptible to various issues that can compromise the safety of bridge operations. Therefore, it is imperative for the highway department to prioritize research on underwater bridge piers’ detection technology. This research enables the timely detection and assessment of problems in underwater bridge piers, allowing for necessary repairs and reinforcements. Inspection techniques such as sonar and diving equipment can be utilized to conduct comprehensive evaluations of structural integrity, load-bearing capacity, and durability. The results of these inspections inform repair and reinforcement efforts, thereby ensuring the normal operation of the bridge and traffic safety.

Due to the challenging environment of underwater bridge piers, conventional methods for bridge pile detection, such as ultrasonic pulse detection, drilling coring, and low-strain methods, are difficult to apply effectively. Instead, various methods based on detecting the geometric characteristics of surface defects are commonly used. These methods include artificial diving/underwater optical imaging technology [1,2], underwater laser imaging technology [3], and underwater sonar imaging technology [4,5].

In the field of underwater detection, the underwater sonar imaging technology has significant advantages compared to other techniques [6]. The underwater sonar imaging technology employs a sonar array to emit sound pulses into the water. When the sound pulse encounters a detection object, it generates a reflected echo. By receiving and analyzing the information from the reflected echo, one can determine the distance between the detection object and the sonar and generate corresponding images. Compared to optical imaging technology, underwater sonar imaging technology has a longer sound wave wavelength (in the megahertz range), weaker scattering, slower propagation, and attenuation in water. It provides a broad imaging range and is unaffected by illumination [7]. These advantages make underwater sonar imaging technology a critical method for underwater bridge detection.

Currently, underwater sonar imaging technology is widely used in various fields, including engineering structure inspection [4,5], underwater pipelines [8,9], seabed topography [10,11], and riverbed exploration. In the context of underwater bridge inspection, relevant research and applications have been conducted. De et al. [12] conducted experiments on bridge scour conditions to study riverbed evolution. Topczewski et al. [13] performed the sonar scanning and imaging of underwater bridge piers and nearby riverbeds, obtaining information on pier outlines and adjacent riverbeds through acoustic image processing. Clubley et al. [14] conducted sonar imaging scans of elevated bridge piers and identified multiple defects that divers had not detected, indicating a higher reliability of sonar detection compared to diver inspections. Hunt et al. [15] analyzed the impact of scour on bridges and developed scour monitoring systems using sonar devices.

Meanwhile, three-dimensional (3D) reconstruction technology uses modern measurement techniques to acquire 2D data and, through computer image processing, reconstructs 3D models that include detailed surface information [16]. Three-dimensional imaging sonar technology offers more detailed descriptions and stereoscopic positioning, providing significant advantages over traditional 2D sonar. Typically, this technology involves a series of processes such as data acquisition, image preprocessing, registration and fusion, and the generation of three-dimensional surfaces. It has a wide range of applications in fields like cultural heritage preservation [17], clinical medicine [18], architectural modeling [19], game development [20], and film special effects [21]. Davis et al. [22] used the Coda Echoscope 3D sonar for high-speed underwater inspections in ports and harbor security, generating real-time 3D images of underwater scenes. Other studies include sonar scanning and imaging detections of railway bridge substructures, dock piles, and bridge foundation scour. Regarding the 3D reconstruction of underwater bridge piers, it can be broadly classified into two categories of methods: model-driven and data-driven. Model-driven approaches involve matching known 3D model information with data obtained from scanning to generate authentic 3D models. In contrast, data-driven methods directly generate planar images from the scanned data and subsequently combine these planar images to construct 3D models. Model-driven methods are suitable for relatively simple structural models, exhibiting generally moderate effectiveness for complex structural models. Conversely, data-driven methods demand a higher data quality but can achieve 3D reconstruction of complex structures.

In conclusion, in the field of underwater bridge pier detection, while many scholars have devoted considerable effort and made significant progress, there are still several pressing challenges. For example, interpreting sonar images depicting surface defects on underwater bridge piers can prove to be quite daunting, especially for individuals lacking a background in sonar technology. Relying solely on a single 2D sonar image for diagnosing these surface defects is often insufficient. Instead, a comprehensive diagnosis necessitates the integration of multiple 2D sonar images captured from various angles. Moreover, it requires inspection personnel to possess a fundamental understanding of sonar imaging principles and to draw upon their expert experience to render accurate diagnoses. This situation bears some resemblance to the way medical doctors must grasp the fundamental principles of CT imaging and rely on their professional expertise to make precise diagnoses based on CT scans of patients.

The sonar image data obtained from single-beam scanning is relatively sparse and of lower accuracy. Additionally, practical engineering constraints limit the range of observation angles, and underwater bridge piers are relatively simple. Therefore, to quickly achieve a 3D model with sufficient engineering accuracy, it seems more appropriate to combine known pier dimensions and use the model-driven approach in this situation. In this approach, we propose a methodology that involves matching the sonar images acquired through scanning with established bridge pier models. We collaboratively reconstruct the underwater bridge piers by combining information sourced from multiple measurement points within the sonar images. Compared to Reference [4], this approach capitalizes on the correspondence between 2D sonar image features of surface defects and 3D shapes, facilitating the precise reconstruction of 3D models depicting surface defects on underwater bridge piers. Subsequently, we use triangular mesh techniques to reconstruct the topology of underwater bridge piers, enabling an approximation of the representation of underwater bridge piers’ surface information. Additionally, we develop specialized digital reconstruction system software tailored for this purpose.

This paper is organized as follows: Section 2 presents an introduction to the mechanism underlying the 3D reconstruction of sonar images of underwater bridge piers. Section 3 introduces an automated recognition method specifically tailored for extracting surface profiles from 2D sonar images. Section 4 focuses on the algorithm employed for the 3D reconstruction of underwater bridge piers. It revolves around the reconstruction of cross-sections of the piers and the generation of a 3D model of the undamaged pier using known information such as dimensions and sonar distance. The identified contour coordinates serve as control points, significantly enhancing the accuracy of the reconstruction process. Section 5 facilitates digital analysis and the observation of surface defects in the 3D models, enabling actions such as 360° rotation, multi-angle viewing, and digital management. This section also discusses how the 3D reconstruction algorithm contributes to the foundational data necessary for 3D model visualization. Section 6 provides a detailed description of the underwater sonar detection experiments conducted on a pier model with man-made surface defects. The reconstructed pier model is subsequently compared with actual measurements to validate the proposed reconstruction and visualization techniques.

## 2. Mechanism of 3D Reconstruction of Sonar Images of Underwater Bridge Piers

To achieve the 3D reconstruction of surface defects on underwater bridge piers using sonar images, it is evident that relying solely on the 2D information derived from individual sonar images of pier contours is insufficient. This limitation becomes particularly pronounced when dealing with underwater bridge piers that exhibit surface defects. Therefore, it becomes imperative to integrate data obtained from multiple sonar image acquisition points and combine them to facilitate recognition and reconstruction. During the fusion and reconstruction process, it is crucial to delve deeper into understanding how information gathered from multiple sonar image acquisition points synergistically contributes to the generation of a 3D model of the underwater bridge piers. This section will begin with an introduction to the imaging principles underlying the MS1000 sonar. Subsequently, it will provide a comprehensive explanation of the mechanism involved in fusing information originating from multiple sonar image acquisition points. It will delve into the analysis of the correspondence between the 2D characteristics of various surface defects within sonar images and their corresponding 3D shapes, presenting potential scenarios. By harnessing the 3D reconstruction mechanism that relies on multiple sonar image acquisition points, we can achieve a more precise determination of potential scenarios, thereby enabling a more accurate reconstruction of the 3D model representing surface defects.

### 2.1. The MS1000 Mechanical Scanning Imaging Sonar

The MS1000 mechanical scanning imaging sonar, proudly crafted by the Canadian company Kongsberg, stands out as a global leader in sonar technology due to exceptional performance characteristics, characterized by precision and resolution.

**Figure 1:**
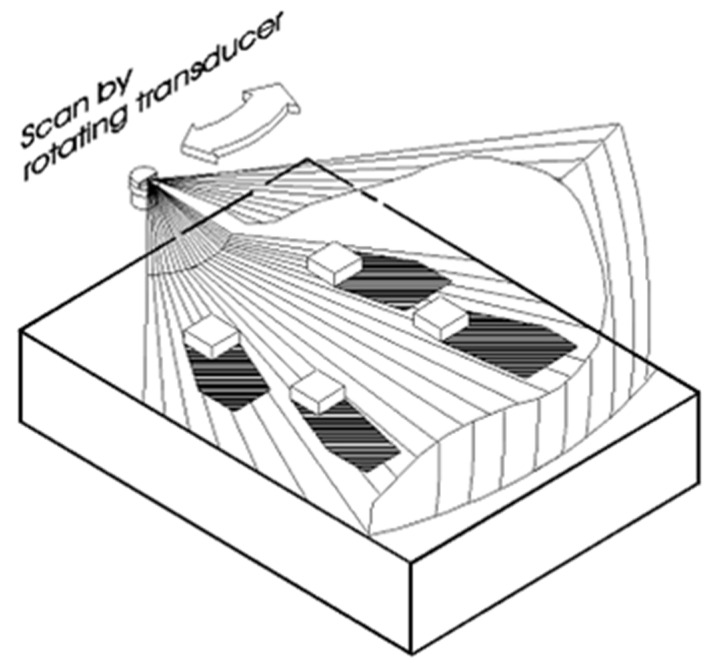
Schematic diagram of acoustic imaging.

As a representative of active sonar systems, the MS1000 sonar possesses the capability to generate sonar images by analyzing echo data. At its core lies the transducer, a vital element within the sonar system. This transducer emits sonar beam pulses into the submerged target area following mechanical rotation to a specific angle. These pulses interact with underwater targets, subsequently reflecting back to the sonar system, thereby generating echo signals. The MS1000 sonar effectively processes and analyzes these echo signals to produce high-quality sonar images, capturing the characteristics and structures of the underwater environment. Notably, the standout feature of the transducer is its ability to achieve a maximum scanning range of 360°, as depicted in Figure 1’s schematic diagram of single-beam scanning sonar.

Figure 1 elucidates the fundamental principles of sonar imaging, where the sonar transducer, when rotated to a specific angle, begins to receive and process reflected waves from identified targets. The collected information correlates with the intensity of the reflected waves, ultimately manifesting as color variations within the sonar image. Regions with higher reflected wave intensities display vibrant colors, while those with lower intensities appear darker. Through a 360° scanning rotation, the sonar transducer constructs a comprehensive sonar image.

Single-beam scanning imaging sonar operates in distance, azimuth, and intensity modes, generating 2D planar images of the underwater environment. While this sonar device is adept at detecting 3D objects, its primary function remains the production of planar images, and it does not provide complete 3D information.

### 2.2. Mechanism of 3D Reconstruction Using Multi-Point Sonar Imaging

**Figure 2:**
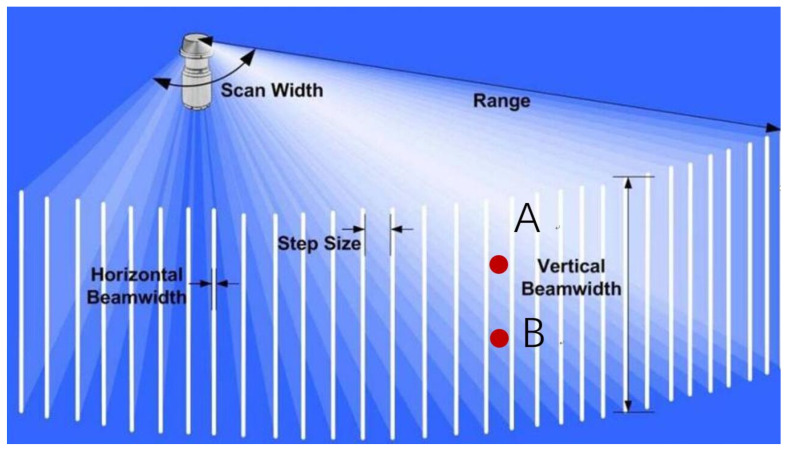
Schematic diagram of single-beam scanning imaging sonar scan.

To achieve precise 3D reconstructions of bridge piers and explore the connection between 2D sonar image features and 3D structures, we initiate by establishing a mapping relationship between the 2D sonar image data and the actual scanning space. As illustrated in Figure 2, we define a spherical coordinate system, denoted as ρθφ, within the spatial domain, with the sonar’s position serving as the origin. Key parameters encompass the sonar’s detection range, denoted as “Range”, the present sonar azimuth angle, represented as θ, the current sonar elevation angle, designated as φ, and the sample count for each beam, referred to as “NumBins.” The equation characterizing the spatial region associated with the *n*th sample point *b* in spherical coordinates is succinctly described as Equation (1).

(1) $$\left\{\begin{array}{c} \theta -0.45{}^{\circ}\leq \theta_{b}\leq \theta +0.45{}^{\circ}\\ \varphi -15{}^{\circ}\leq \varphi_{b}\leq \varphi +15{}^{\circ}\\ Range\times \frac{\left[\rho \times \frac{NumBins}{Range}\right]}{NumBins}\leq \rho_{b}\leq Range\times [\rho \times \frac{NumBins}{Range}+1]/NumBins \end{array}\right.$$

Sonar images encapsulate temporal, angular, and intensity data. It is noteworthy that for data points where different angles correspond to identical time-of-arrival instances, such as points A and B (See Figure 2), the sonar is incapable of distinguishing between them.

**Figure 3:**
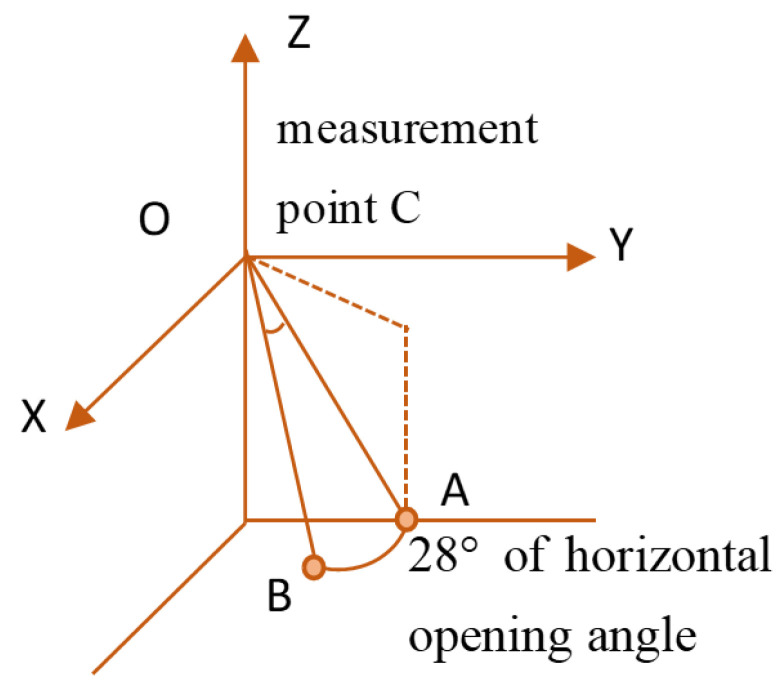
Schematic diagram of imaging principle.

Figure 3 illustrates that when the sonar is positioned at measurement point C_1_, the transducer emits sonar beam pulses towards the underwater bridge pier region. Points A and B reside on the same beam arc, and at the peak of reverse-scattered echo intensity along the direct linear distance, they concurrently reach the sonar. In this scenario, the sonar cannot differentiate between points A and B, leading to their coalescence in the sonar image, manifesting as a single pixel. Nonetheless, when the sonar scans from an alternative measurement point, C_2_, points A and B occupy disparate beam pulse arcs, causing variations in their arrival times at the sonar. Consequently, the sonar distinguishes between points A and B, representing them as distinct pixels in the sonar image. Analogously, points that are indistinguishable when the sonar is positioned at measurement point C_2_ can typically be discerned when positioned at measurement point C_1_. Leveraging this complementary relationship between measurements at points C_1_ and C_2_ facilitates the derivation of 3D information concerning the underwater bridge piers.

**Figure 4:**
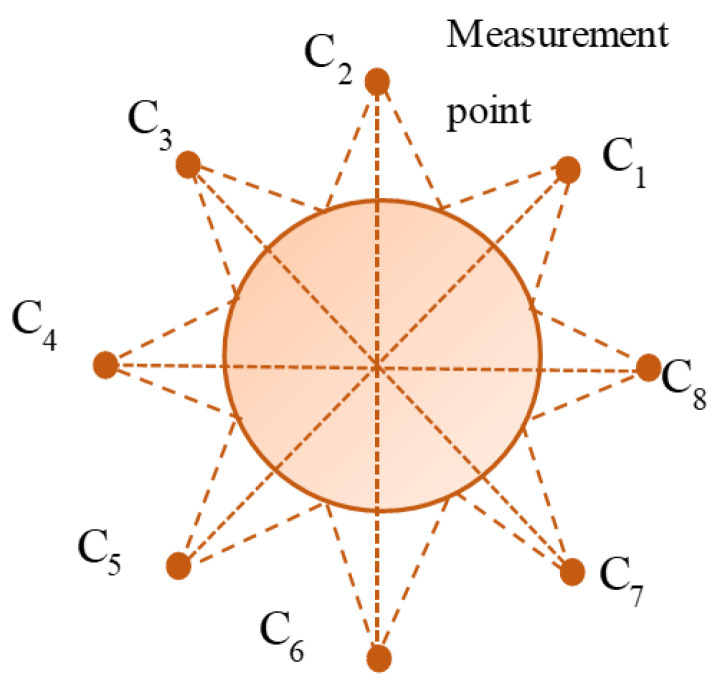
Schematic of measurement point beam coverage area.

When conducting sonar scanning imaging on cylindrical bridge piers, a multi-point approach is utilized to generate multiple 2D sonar images, as illustrated in Figure 4. Eight designated measurement points, labeled C_1_, C_2_, …, C_8_, are strategically positioned. The dashed lines in the figure represent the emitted beam pulses originating from measurement points C_1_, C_2_, …, C_8_. By performing the multiplication of intensity data corresponding to pixels at the intersection of these two beam pulses, a dataset is generated. If a specific region is covered by multiple beam pulses and exhibits a heightened reflection intensity, the result of the multiplication will increase, indicating the presence of an underwater bridge pier in that region. Conversely, if a region displays a substantial reflection intensity on only one beam pulse while the other beam pulse indicates no significant reflection intensity, the result of the multiplication will decrease, implying the absence of an underwater bridge pier in that area. Consequently, the determination of underwater bridge pier presence or absence can be made based on these multiplication values.

**Figure 5:**
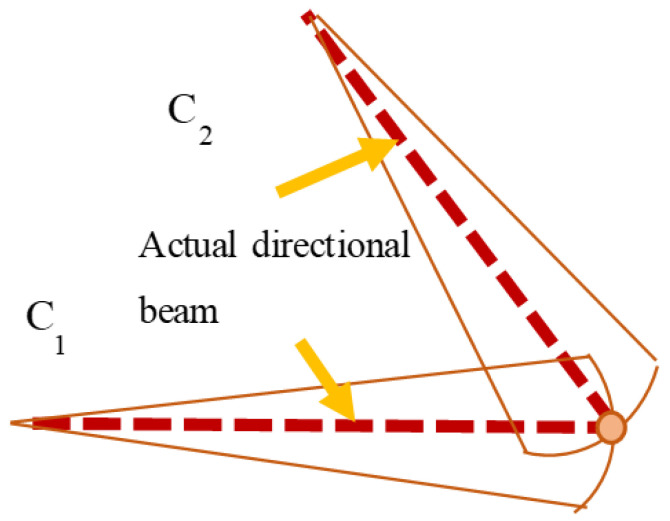
Schematic of actual target point directional beam.

In simpler terms, this means that if the sonar image data acquired from one measurement point are converted into 3D spatial data and then multiplied with 3D spatial data derived from sonar image data obtained from another measurement point, and both results demonstrate a high reflection intensity, they will form spatial data with peak regions. This facilitates the accurate spatial localization of underwater bridge piers. By adopting this method, the actual direction of the beam pulse can be ascertained using data obtained from two sonar images, as depicted in Figure 5.

Hence, by multiplying sonar image data from multiple measurement points, along with utilizing pre-existing knowledge of undamaged bridge pier models and the established correspondence between specific surface defect features in 2D sonar images and their 3D morphologies, it becomes feasible to achieve the 3D reconstruction of underwater bridge piers afflicted with surface defects.

## 3. Automated Recognition of 2D Sonar Images for Underwater Bridge Piers Surface Profile

**Figure 6:**
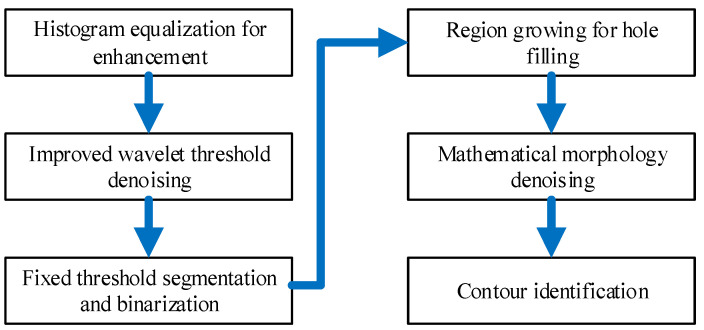
Flowchart of automated recognition of 2D sonar images for underwater bridge piers surface profile.

The recognition of underwater bridge piers’ surface profiles plays a pivotal role in the field of 3D digital reconstruction, as it directly influences key factors such as efficiency, complexity, and precision in the 3D digital reconstruction process. In this section, we introduce an automated recognition method designed to extract underwater bridge pier surface profiles from 2D sonar images. The workflow for this method is visually depicted in Figure 6, and it encompasses the following primary steps:

(1) Histogram equalization for enhancement: As an initial step, we employ the histogram equalization method to broaden the grayscale range of sonar images with the goal of enhancing the data. This step is undertaken to bolster image contrast and quality.

(2) Improved wavelet threshold denoising: Subsequently, we apply an enhanced wavelet threshold denoising function to the sonar images. This step serves to mitigate noise in the images and elevate the accuracy of subsequent processing stages.

(3) Fixed threshold segmentation and binarization: We employ a fixed thresholding approach to segment underwater bridge piers information and subsequently convert it into binary images, thus facilitating superior differentiation between targets and the background.

(4) Region growing for hole filling: To ensure the integrity of the bridge piers’ information, we implement a region-growing method to autonomously fill gaps within the sonar images.

(5) Mathematical morphology denoising: Further refinement of image quality is achieved through the use of mathematical morphology operations designed to eliminate protrusions and indentations along the edges of bridge piers.

(6) Contour identification: Finally, we leverage the findContours function to identify the surface profile of underwater bridge piers, extracting both the inner and outer contour information of the piers and the upper and lower contour data of the riverbed.

In summary, we have successfully integrated this methodology into digital detection software, resulting in the automated recognition of 2D sonar images for underwater bridge piers’ surface profiles.

**Figure 7:**
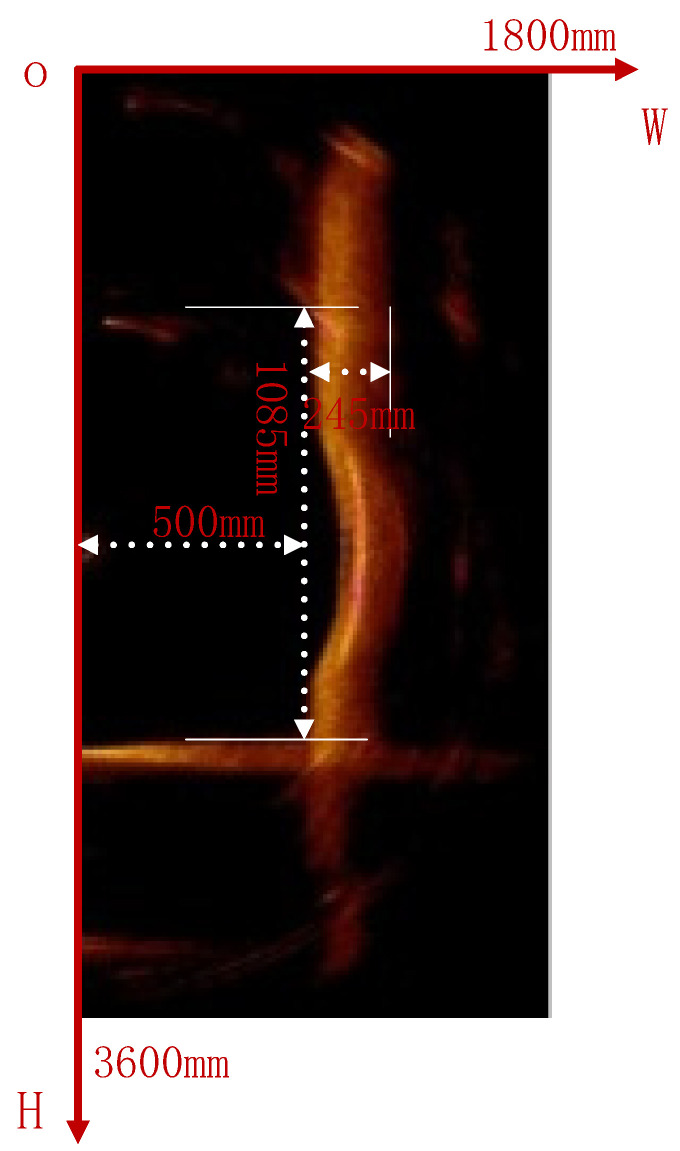
Original pseudo-color sonar image of an underwater bridge pier.

In this section, we illustrate the automated recognition process of the 2D sonar image for the surface profile of underwater bridge piers using an example of original pseudocolor sonar images of underwater bridge piers. The dimensions of the original pseudocolor sonar image of underwater bridge piers are 450 pixels by 900 pixels, with the sonar positioned 500 mm away from the center of the pier, and the underwater portion of the pier measuring 1085 mm in length and 245 mm in diameter, as depicted in Figure 7.

### 3.1. Histogram Equalization for Enhancement

Sonar images are typically grayscale images, and their grayscale information plays a crucial role in the intelligent recognition of surface anomalies and 3D digital reconstruction. Histogram equalization is a process that involves statistically analyzing grayscale values in sonar images to remap them into a uniform distribution. This process stretches and compresses the grayscale levels of the image, thereby expanding the dynamic range of grayscale values to achieve data enhancement [23].

Let f and g represent the original image and the image after histogram equalization, respectively, with image dimensions of m × n and a grayscale value range of 0 to 255, i.e., L = 256. The steps for histogram equalization are as follows:

(1) Calculate the grayscale histogram of the sonar image f, denoted as h.

(2) Calculate the cumulative grayscale distribution frequency v(μ), defined by the following formula:

(2) $$v(\mu )=\sum_{k=0}^{\mu }\frac{h(k)}{m\times n}$$

Here, h(k) represents the number of pixels with the k-th grayscale level, and m×n represents the image size.

(3) Determine the minimum cumulative grayscale distribution frequency vmin.

(4) Calculate the grayscale values ω for g using the following formula:

(3) $$\omega =\left\lfloor \left(L-1)(\frac{v\left(\mu \right)-v_{min}}{1-v_{min}}\right)\right\rfloor$$

Here, ⌊ ⌋ represents floor rounding.

**Figure 8:**
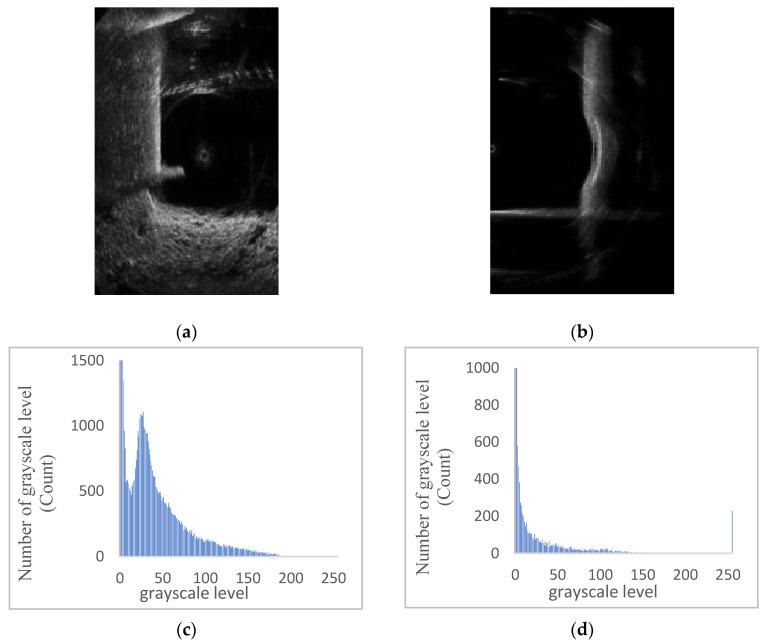
Histogram statistical examples of partial bridge pier sonar grayscale images. (**a**) Grayscale sonar image of actual bridge pier. (**b**) Grayscale sonar image of bridge pier experimental model. (**c**) Actual bridge pier sonar grayscale image histogram. (**d**) Bridge pier experimental model sonar grayscale image histogram.

Insufficient contrast in an image is often manifested by a concentration of the pixel values in the histogram. Images with lower illumination tend to have their histogram components concentrated in the low grayscale levels, while images with higher illumination and distinct grayscale tend to have their histogram components concentrated in the high grayscale levels. Figure 8 illustrates an example of grayscale histogram statistics after grayscale processing for some original pseudocolor sonar images. From Figure 8, it is evident that the grayscale distribution of underwater bridge piers sonar images is uneven. Hence, it is necessary to apply local histogram equalization.

**Figure 9:**
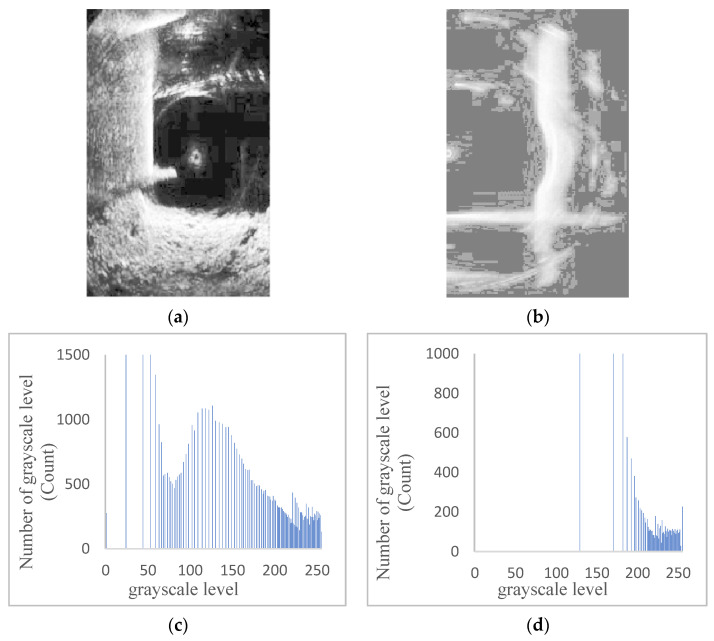
Results after histogram equalization. (**a**) Processed grayscale image of actual bridge pier. (**b**) Processed grayscale image of bridge pier experimental model. (**c**) Processed histogram of actual bridge pier. (**d**) Processed histogram of bridge pier experimental model.

Figure 9 displays the results after applying histogram equalization. Through this process, the contrast of sonar images is significantly enhanced, the grayscale distribution range is widened, and the bridge piers’ contour information becomes clearer.

### 3.2. Improved Wavelet Threshold Denoising

Sonar images are a type of vector signal, and thus, spatial domain filtering methods like mean filtering and median filtering have limited effectiveness in removing noise from such stationary random vector signals [24].

In this section, we have improved and combined the Garrote function and soft threshold function to create an enhanced wavelet threshold denoising function, which can be mathematically expressed as follows:

(4) $${\hat{d}}_{j,k}=\left\{\begin{array}{c} d_{j,k}-m\lambda sgn\left(d_{j,k}\right)-\left(1-m\right)\frac{\lambda^{2}}{d_{j,k}},\;\left|d_{j,k}\right|\geq \lambda ,\;\;m\in [0,1]\\ 0,\;\;\;\left|d_{j,k}\right|<\lambda \end{array}\right.$$

Here, the parameter “m” is used for adjustment. The enhanced function possesses the characteristics of both the soft threshold function and the Garrote function. When *m* = 1, it takes the form of a soft threshold function, and when *m* = 0, it resembles the Garrote function. When *m* falls within the range of (0, 0.5), the enhanced function tends to exhibit the characteristics of the Garrote function, resulting in image smoothing while preserving edge features. When *m* falls within the range of (0.5, 1), the enhanced function leans more towards the soft threshold function, with a more continuous function curve, leading to smoother images. The improved wavelet threshold denoising function combines the advantages of the Garrote function and the soft threshold function, allowing for flexible selection of the parameter “m” based on the denoising requirements.

**Figure 10:**
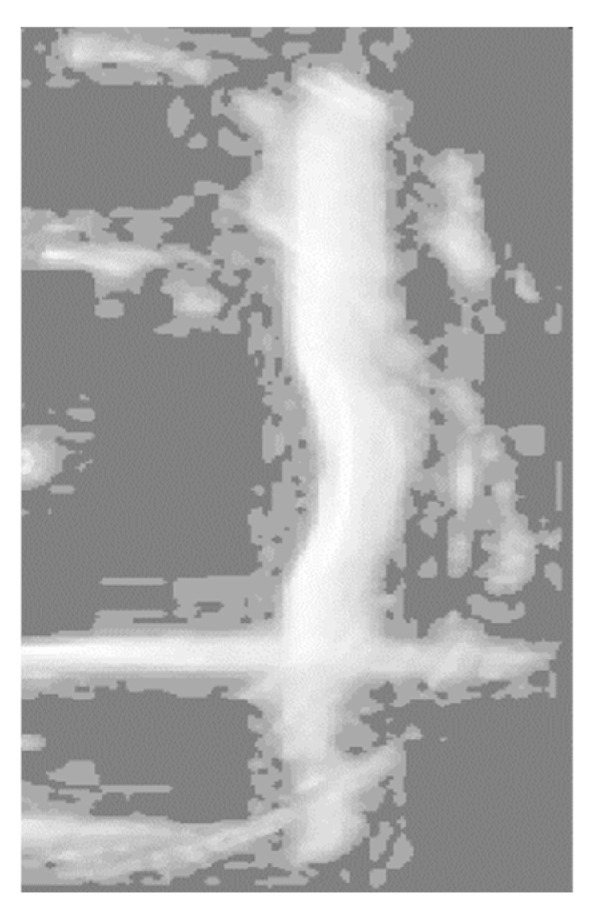
Filtering of sonar image.

According to research findings, the use of the improved wavelet threshold denoising function in filtering, compared to basic mean filtering, basic median filtering, and traditional wavelet threshold denoising methods, results in the smallest mean squared error and the highest signal-to-noise ratio and peak signal-to-noise ratio. Therefore, this study employs this filtering method for denoising. As shown in Figure 10, the denoised results are presented, clearly illustrating that the denoised image is smoother compared to the original image.

To validate the denoising effectiveness of the improved wavelet threshold function, this paper objectively compares and analyzes various denoising methods using empirical data. Initially, Gaussian white noise is added to sonar images of underwater bridge piers. Subsequently, denoising techniques are applied to these noisy sonar images, followed by numerical evaluation using the mean square error (MSE), peak signal-to-noise ratio (PSNR), and signal-to-noise ratio (SNR). The denoising methods evaluated include basic mean filtering, basic median filtering, as well as hard thresholding, soft thresholding, Garrote function, and the improved wavelet threshold function for wavelet denoising. Evaluation metrics are summarized in Table 1. Analysis of Table 1 reveals that the improved wavelet threshold function achieves the lowest MSE and highest SNR and PSNR values, demonstrating a superior denoising performance. Consequently, this paper adopts the improved wavelet threshold function for preprocessing image denoising.

### 3.3. Fixed Threshold Segmentation and Binarization

**Figure 11:**
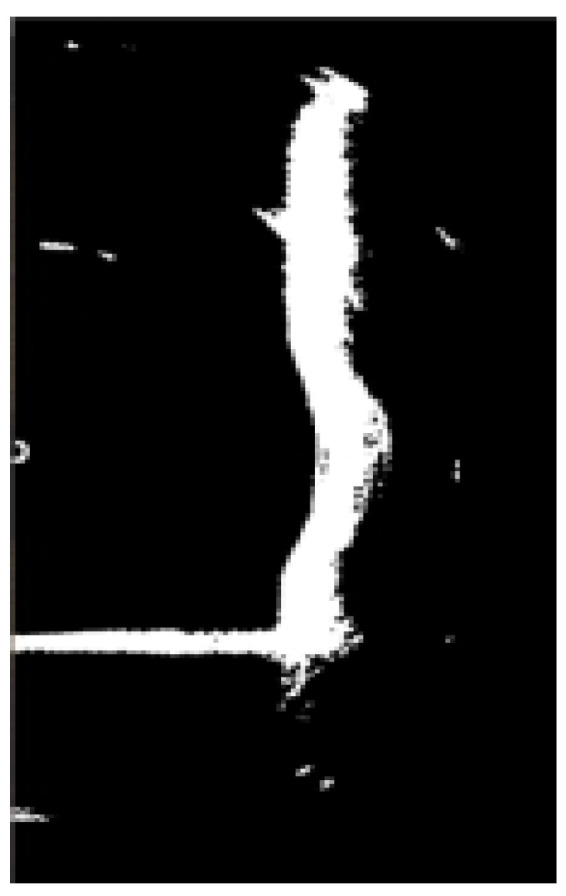
Binarization of sonar image.

Following the previous histogram equalization process, the histogram exhibits a bimodal distribution, representing two distinct object classes with significant grayscale differences. This corresponds to an image consisting of a darker background and brighter targets, as depicted in Figure 9d. By carefully selecting an appropriate threshold value “T” at the trough point between the two histogram peaks, it becomes possible to effectively separate the bridge pier from the background. Consequently, for image segmentation in this section, we have opted for the fixed threshold method, configuring “T” to 220. Simultaneously, the image undergoes binarization, where pixels less than or equal to the threshold are assigned a grayscale value of zero, while pixels exceeding the threshold are assigned a grayscale value of 255. Figure 11 illustrates the results of applying the fixed threshold segmentation to Figure 10.

By comparing Figure 10 and Figure 11, it becomes evident that after the process of image segmentation and binarization, the image presents a distinct black-and-white effect. This effectively accentuates the contour information of the underwater bridge pier while faithfully representing the mirrored reflection of the underwater pier.

### 3.4. Region Growing for Hole Filling

Due to the uneven surface of the bridge pier, non-reflective areas exist on its surface. Within the bridge pier region, dark areas with grayscale values lower than the ideal threshold are present. Furthermore, due to noise and the reflection and refraction of the water surface, white regions appear outside the bridge pier area, as depicted in Figure 11. Achieving precise recognition necessitates filling these data gaps and removing both the dark and white regions. This task is accomplished using a region-growing method [25].

**Figure 12:**
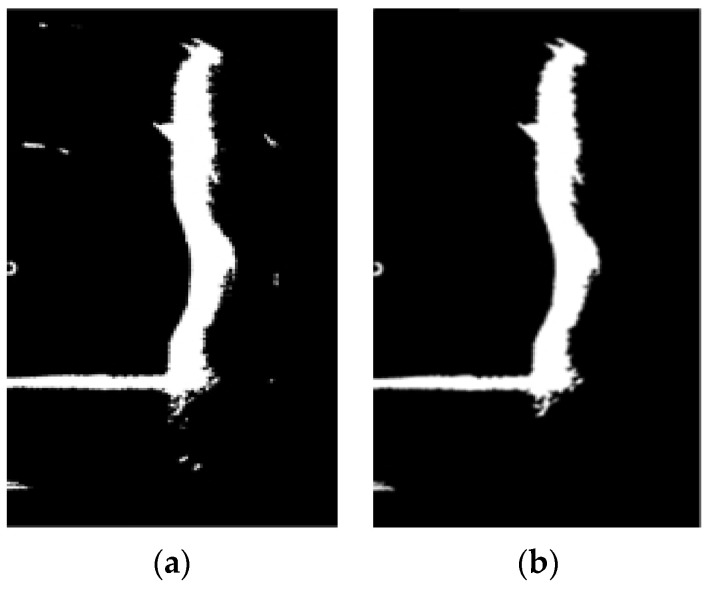
Removal of black and white regions in sonar image. (**a**) Sonar image after black region removal. (**b**) Sonar image after white region removal.

Specifically, for the removal of dark regions in the underwater bridge piers’ sonar image, the entire image is scanned. White pixels are considered valid, and a 4-neighbor detection is utilized to calculate the number of pixels in each region. If the total count falls below a predetermined threshold, the region is cleared, effectively filling the dark regions, as illustrated in Figure 12a. When eliminating white regions, a subsequent scan of the entire image is conducted, with black background pixels being deemed valid. An 8-neighbor detection scheme is employed to compute the pixel count in each region. If the total count falls below a predefined threshold, the region is cleared, resulting in the filling of white regions, as shown in Figure 12b.

Upon comparing Figure 12a,b, it becomes evident that by removing the dark and white regions from the sonar image, extraneous noise information is further eliminated. The sonar image now exclusively retains the underwater bridge pier area, consequently reducing computation time for subsequent defect extraction and 3D digital reconstruction processes.

### 3.5. Mathematical Morphology Denoising

Despite the removal of dark and white regions, the edges of the bridge pier still exhibit numerous jagged protrusions, leading to elongated edge contours. These protrusions increase the computational complexity during subsequent contour recognition and can potentially cause misidentification. To address this issue, mathematical morphology operations, such as erosion and dilation, are commonly employed to smooth out these jagged features along the bridge pier’s edges.

Mathematical morphology serves as a valuable tool for nonlinear image (signal) processing and analysis, with its primary focus on the geometric structure and interrelationships within images. By employing various “structuring elements”, it is possible to measure or extract corresponding shapes and features from the image. Morphological operations encompass erosion and dilation, and a variety of practical morphological algorithms can be developed and combined based on these two fundamental operations. Erosion and dilation operations in binary images are dual operations. The erosion of X by S, denoted as X⊝S, is defined as the set of all points x for which the translation of S by x is entirely contained within X:

(5) $$X⊝S=\left\{x|S+x\subseteq X\right\}$$

Dilation of X by S, denoted as X⨁S, is defined as the set of all points x for which the translation of S by x has a non-empty intersection with X:

(6) $$A⨁S=\left\{x|S+x\cup x\neq \emptyset \right\}$$

**Figure 13:**
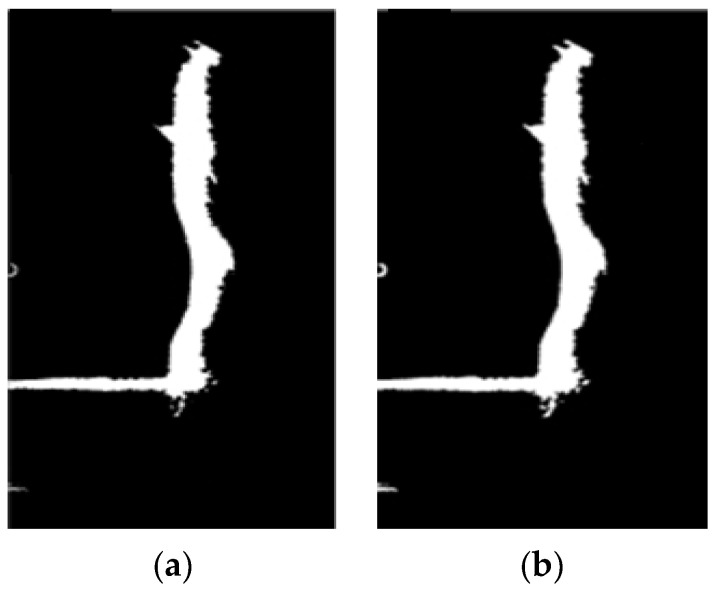
Morphological erosion and dilation. (**a**) Erosion treatment to eliminate edge spikes. (**b**) Dilation treatment to preserve spikes.

In this study, a 3 × 3 square structuring element is employed for further morphological erosion and dilation operations on the binary image, as illustrated in Figure 13. Figure 13a showcases the outcome following the erosion operation. When compared to the binary image in Figure 12b, it is evident that the white isolated noise points along the underwater bridge pier’s edge have been significantly reduced in the sonar binary image after the erosion operation. Figure 13b presents the image following the application of the dilation operation to the image from Figure 13a. In contrast to Figure 13a, the white pixels appear more concentrated, indicating stronger connectivity within the underwater bridge pier’s edge region. Post-processing, when compared to the original image, results in smoother edges with fewer jagged features, thus facilitating subsequent data extraction. Furthermore, a substantial portion of the internal and external noise is eliminated, thereby reducing the potential for misjudgment.

### 3.6. Contour Identification

**Figure 14:**
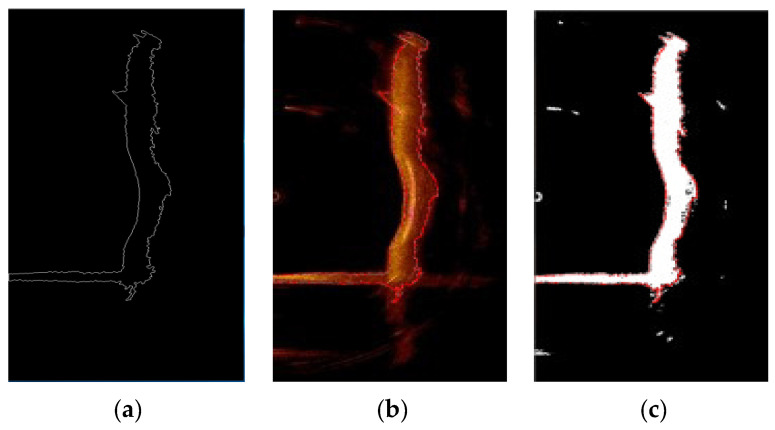
Bridge pier contour recognition. (**a**) Extracted contour information. (**b**) Comparison with the original sonar image. (**c**) Comparison with the binarized sonar image.

To meet the requirements for subsequent 3D reconstruction, it is necessary to further extract the contours of the underwater bridge pier and riverbed. This involves identifying both the inner and outer contours of the bridge pier in the sonar image and the upper and lower contours of the riverbed. The automated steps for sonar image contour recognition are as follows:

(1) In the sonar image, the underwater bridge pier and riverbed form a connected structure and represent the largest target contours in the sonar image. Therefore, the “findContours” function provided by OpenCV is utilized to identify the contours of image regions. The area of each contour is recorded, and the contour with the largest area is identified as the target, which is the bridge pier, as shown in Figure 14a.

(2) Determine the coordinates of the uppermost contour and the lowermost contour.

(3) For the outer contour, trace the next contour coordinate point to the left from the uppermost contour coordinate, continuing until the lowermost contour coordinate is reached.

(4) For the inner contour, trace the next contour coordinate point to the right from the uppermost contour coordinate, continuing until the lowermost contour coordinate is reached.

(5) Repeat the above steps for another sonar image until all sonar images have been processed.

Finally, the extracted underwater bridge pier contour information is overlaid onto the original sonar image and the binary sonar image. To emphasize the contours, they are marked in red, as shown in Figure 14a,b. From Figure 14a,b, it can be observed that the extracted underwater bridge pier contour information closely aligns with the actual contour information, demonstrating the accuracy of this method in extracting underwater bridge pier contours. In the end, all the inner and outer contours of the bridge piers in the sonar images and the upper and lower contours of the riverbed are obtained for subsequent 3D digital reconstruction purposes.

## 4. Underwater Bridge Piers 3D Reconstruction Algorithm

**Figure 15:**
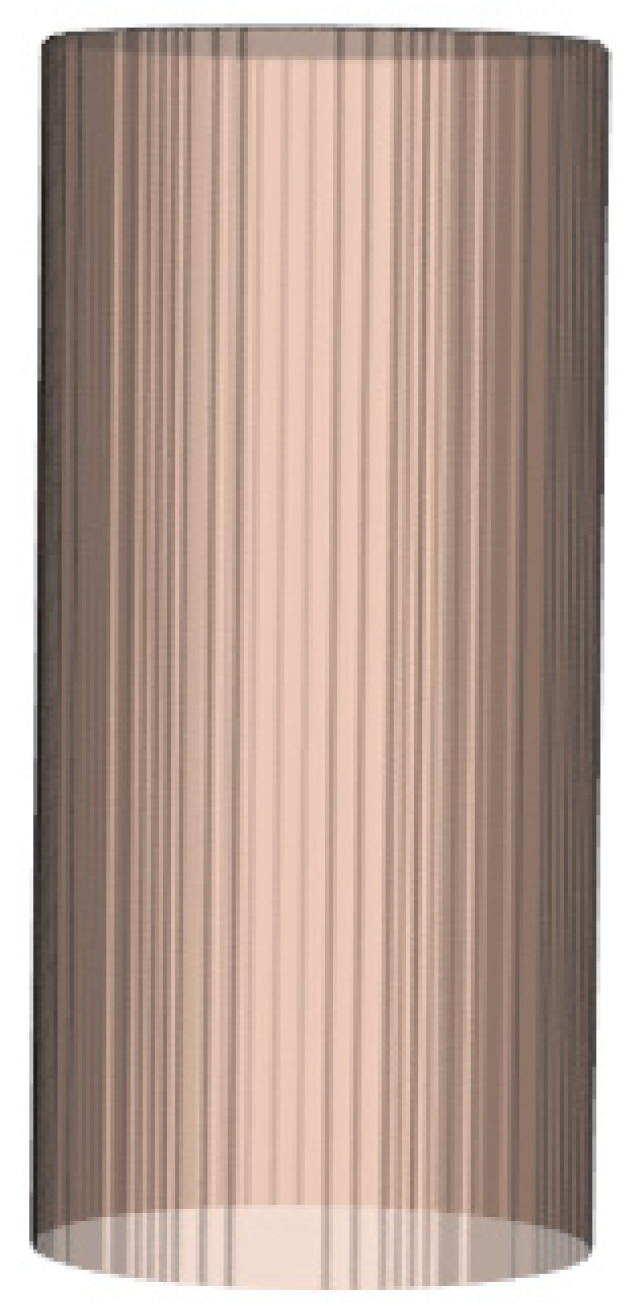
Undamaged bridge pier model.

This algorithm is centered around the reconstruction of underwater bridge pier cross-sections. It generates a 3D model of the bridge pier in its undamaged state, leveraging known information like the pier’s dimensions, the distance between the sonar device and the pier, and more. Furthermore, it utilizes the identified coordinates of the bridge pier’s contours as control points and combines them with the multiplication of multi-point sonar data to further refine potential scenarios. Ultimately, it culminates in the reconstruction of the authentic 3D model of the underwater bridge pier with surface defects. Here are the detailed algorithm steps:

Step 1: Based on actual measurements of the bridge pier’s dimensions, create a three-dimensional array V to represent the undamaged bridge pier’s 3D model. The elements of the array $V_{xyz}$ are defined according to Formula (7), with a visual representation as depicted in Figure 15.

(7) $$\left\{\begin{array}{c} V_{xyz}=255\;when\sqrt{x^{2}+y^{2}}\leq R\;and\;0\leq R\leq H,\;inside\;the\;bridge\;pier\;\;\;\;\\ V_{xyz}=0\;when\sqrt{x^{2}+y^{2}}\geq R\;or\;\;R<0\;or\;R>H,\;outside\;the\;bridge\;pier \end{array}\right.$$

**Figure 16:**
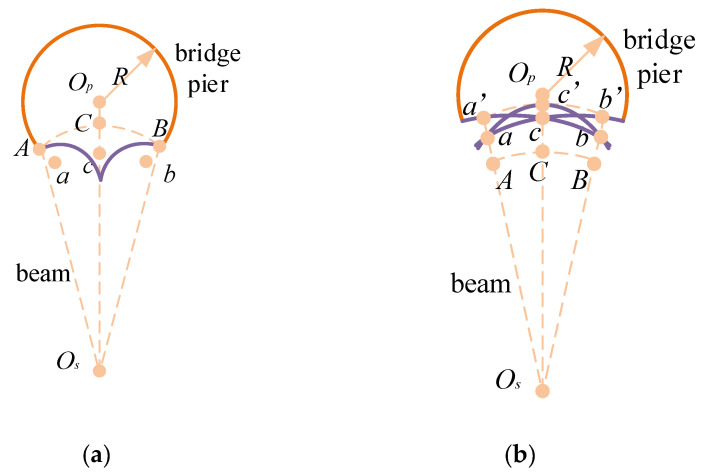
Schematic representation of possible 3D shapes of continuous measurement points. (**a**) Measurement Point 1. (**b**) Measurement Point 2.

Step 2: Set Z to a value m and select $V_{xyz}$ to represent a cross-section of the bridge pier. Proceed to intersect the coordinates of n measurement points with the current cross-section. As illustrated in Figure 16, the aim of this step is to derive potential scenarios for the 3D morphological analysis of continuous sonar images gathered from multiple measurement points, each of which contains information that cannot be ascertained solely from a single-point sonar image. Op is the center of the bridge pier cross-section; *R* is the radius of the bridge pier; Os is the position of the sonar transducer; possible bridge pier profile shapes such as ABC, a’cb, ac’b, acb’ are obtained based on the distance of the profile.

**Figure 17:**
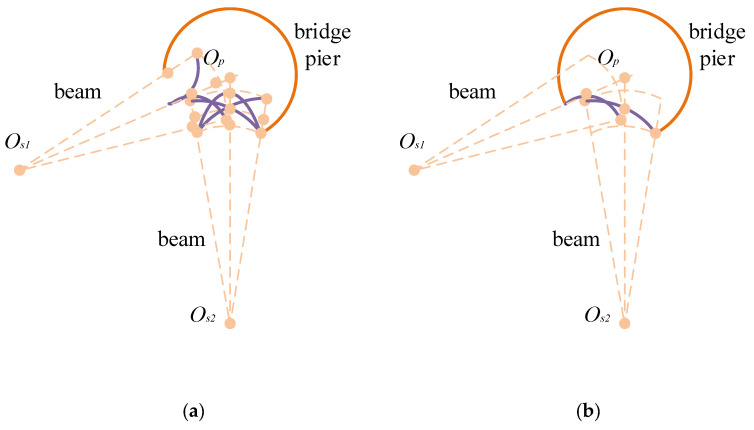

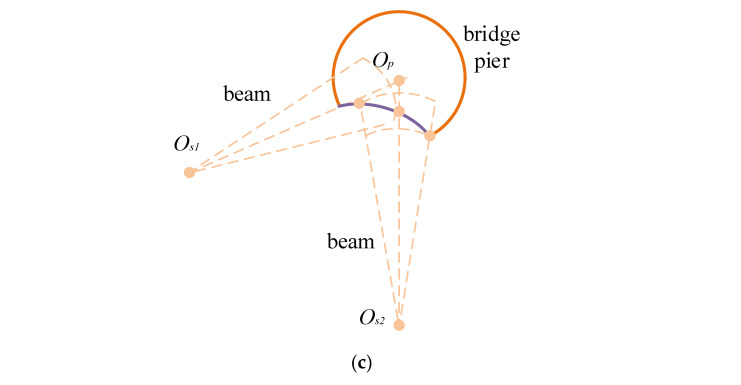
Schematic representation of surface damage reconstruction for adjacent measurement points. (**a**) All control points corresponding to the measurement point. (**b**) Excluded contour. (**c**) Final surface damage contour.

Step 3: Within the current cross-section, perform a multiplicative assessment of the 3D morphological scenarios obtained in Step 2, using data from multiple measurement points. The scenario yielding the highest multiplication result is chosen as the final outcome, and the cross-sectional contour of the current bridge pier is adjusted accordingly. For instance, hole contour reconstruction can be accomplished by utilizing sonar images from neighboring measurement points, as depicted in Figure 16. All key points from Figure 16a and Figure 16b are combined, as displayed in Figure 17a. Then, based on the relative positions of the measurement points, select the surface defect contour on the side close to O_s2_ and the one close to O_s1_, resulting in a contour shape that best matches the key points, as shown in Figure 17b. Ultimately, the constructed surface defect contour is presented in Figure 17c.

**Figure 18:**
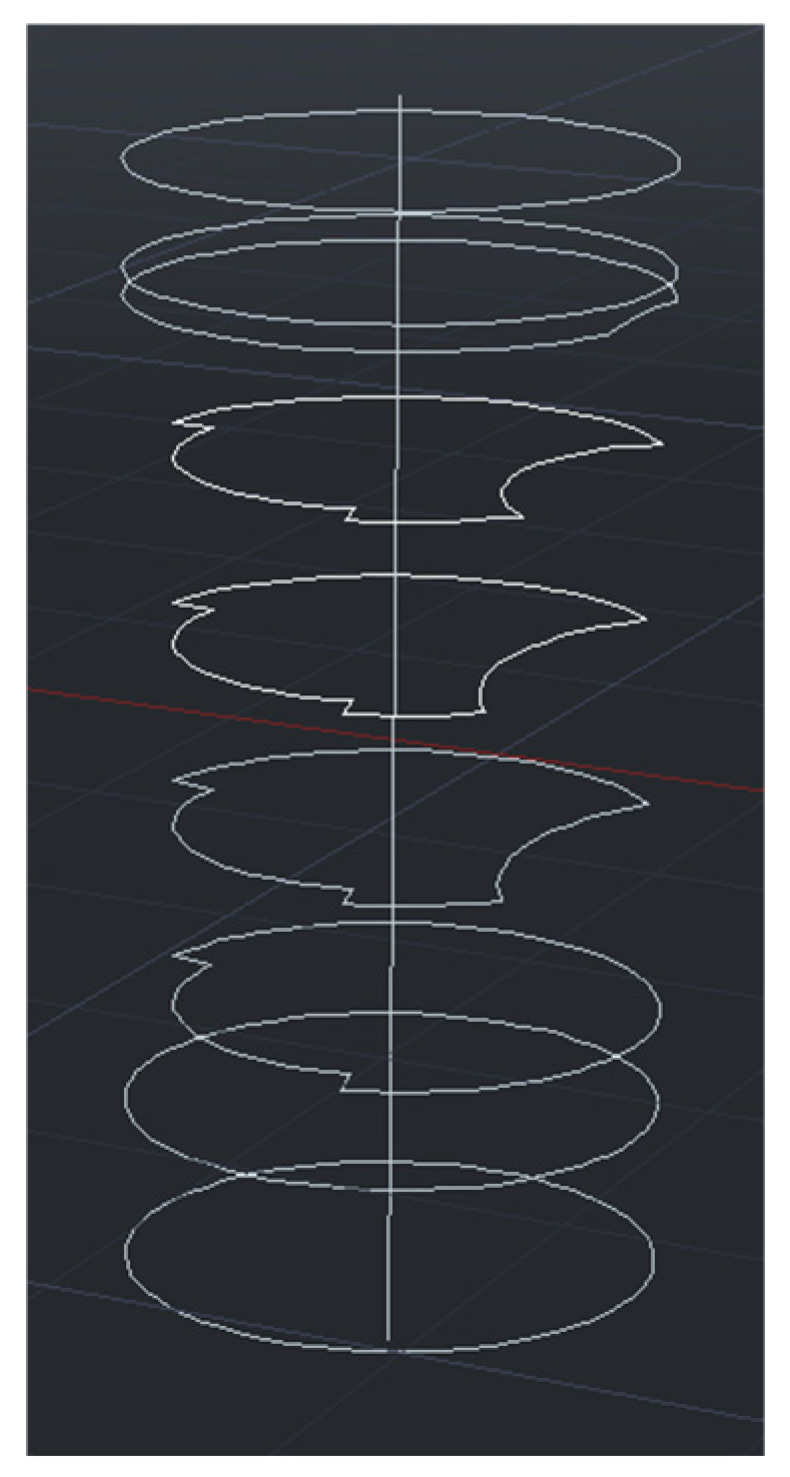
Fitting the bridge piers contour for the current cross-section.

Step 4: Set Z to values 0, 1, …, H, and then repeat Step 2 and Step 3 to obtain the bridge pier contours for all cross-sections. In the end, these contours will be employed for the reconstruction of the 3D model of the bridge pier, as depicted in Figure 18.

## 5. Three-Dimensional Visualization of Underwater Bridge Piers Sonar Images Using VTK

Three-dimensional visualization provides an intuitive and dynamic representation of underwater bridge pier models on a computer, aiding researchers in digitally analyzing surface defects. However, the 3D reconstruction algorithm described earlier focuses primarily on fitting cross-sectional contours and obtaining raw data. These data serve as foundational for 3D model visualization but do not convey detailed 3D model information.

To achieve computer-based 3D visualization of underwater bridge pier models, we utilize the Visualization Toolkit (VTK). This section compares different 3D visualization algorithms, focusing on surface rendering methods for displaying surface defects in the bridge pier model. The visualization program is integrated into digital inspection software.

There are various 3D reconstruction algorithms, broadly categorized into volume rendering and surface rendering. Surface rendering, chosen here for its suitability in handling underwater bridge pier surface defects with reduced computational complexity, employs VTK’s Marching Cubes (MC) algorithm. This approach involves extracting isocontours, reducing triangles, smoothing data, generating normals, and triangulating isocontours to achieve 3D visualization.

**Figure 19:**
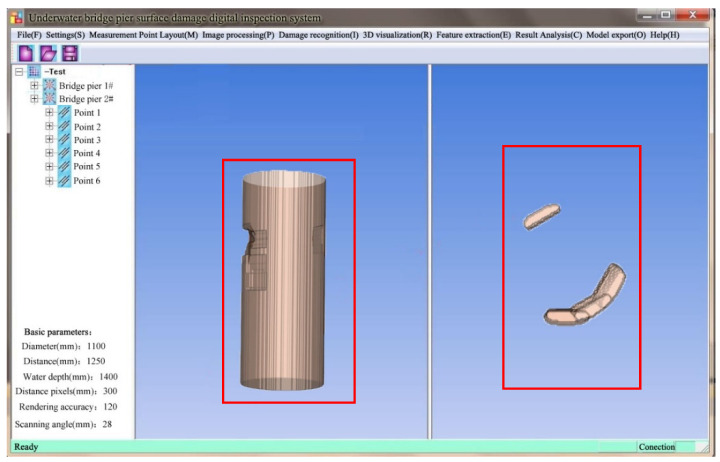
The visualization software.

Furthermore, leveraging sonar image data processing and 3D reconstruction algorithms, we developed the Digital Inspection System Software for Underwater Bridge Pier Surface Defects. This software, developed in C++ with an interface in QT and using VTK for visualization, supports parameter setting, image processing, 3D visualization, feature extraction, result analysis, and model export (Figure 19).

As shown in Figure 19, this software serves as a platform for processing sonar images and 3D reconstruction of underwater bridge piers. It enables 3D visualization of underwater bridge pier models and offers interactive functions such as translation, scaling, and rotation, facilitating the analysis and observation of surface defects in the 3D models of underwater bridge piers by researchers.

## 6. Underwater Bridge Piers’ 3D Reconstruction Experiment

To validate the feasibility and accuracy of the 3D reconstruction technology, this section conducts underwater bridge pier sonar scanning experiments in a controlled environment. The sonar images obtained from the scans are used as input for the 3D reconstruction process. The previously described 3D reconstruction algorithm and visualization methods are employed to reconstruct the 3D model of the underwater bridge pier. Finally, the dimensions of the reconstructed 3D model are measured and compared with the actual model dimensions for analysis and validation.

### 6.1. Experimental Environment

**Figure 20:**
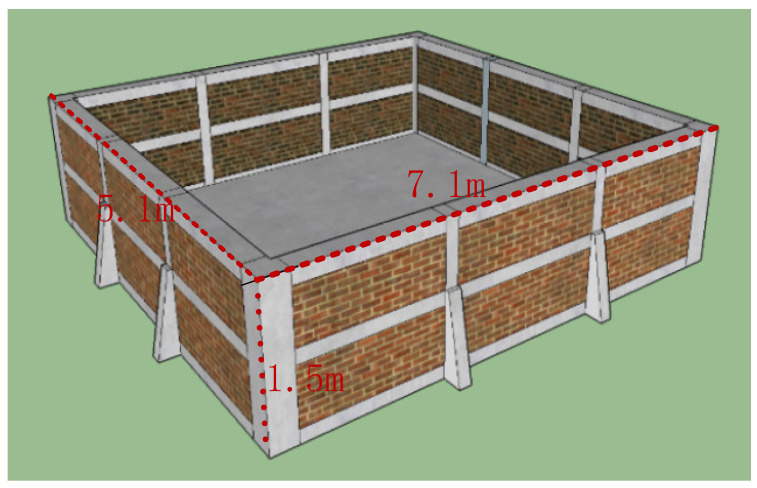
Schematic diagram of the 7.1 m × 5.1 m × 1.5 m water tank structure.

**Figure 21:**
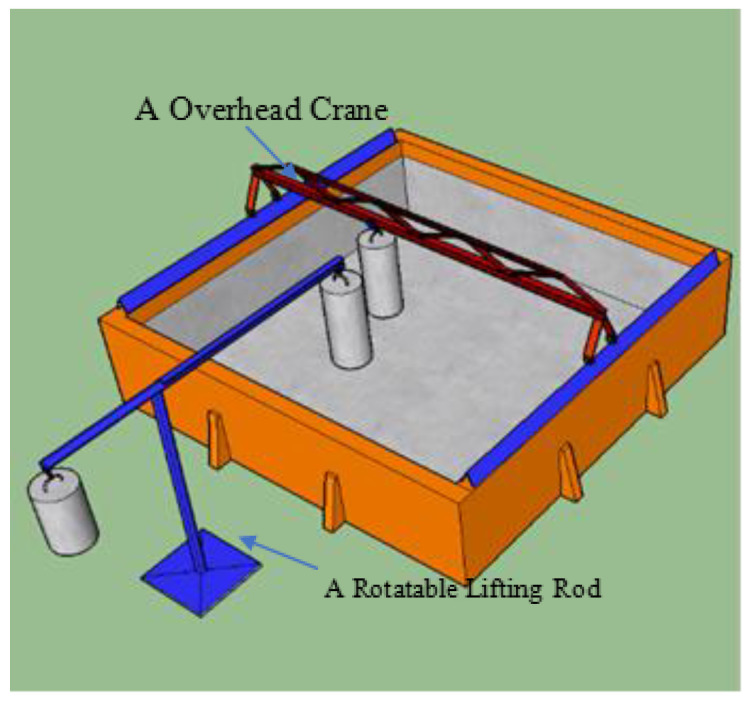
The water tank hoisting system.

A laboratory environment was set up to simulate underwater inspection conditions. A water tank measuring 7.1 m × 5.1 m × 1.5 m was constructed and filled with water, as depicted in Figure 20. To facilitate hoisting large concrete column models, we installed a miniature overhead crane with horizontal movement capabilities and a rotatable lifting rod, as shown in the water tank hoisting system illustrated in Figure 21.

**Figure 22:**
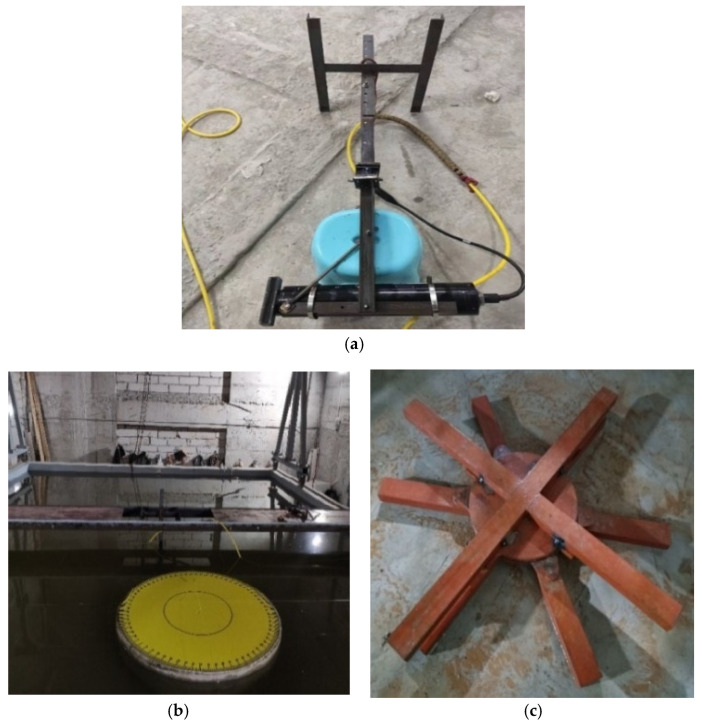
Experimental auxiliary device diagram. (**a**) Schematic of sonar fixed on angle steel frame. (**b**) Test tank and mobile operating platform. (**c**) Underwater turntable.

For the stability and safety of the sonar equipment during inspections, a set of auxiliary devices, depicted in Figure 22, were designed and constructed. This testing auxiliary device includes an angle steel frame, a movable operating platform, and a turntable. The angle steel frame enables adjustments in the sonar angle and height within the water environment, as seen in Figure 22a. The movable operating platform, positioned above the water tank, supports the angle steel frame, allowing its free movement, as depicted in Figure 22b. At the bottom of the tank, a turntable holds the underwater bridge pier model, allowing us to alter the positions of the sonar measurement points by rotating the turntable, as shown in Figure 22c.

### 6.2. Concrete Column Bridge Pier Models

**Figure 23:**
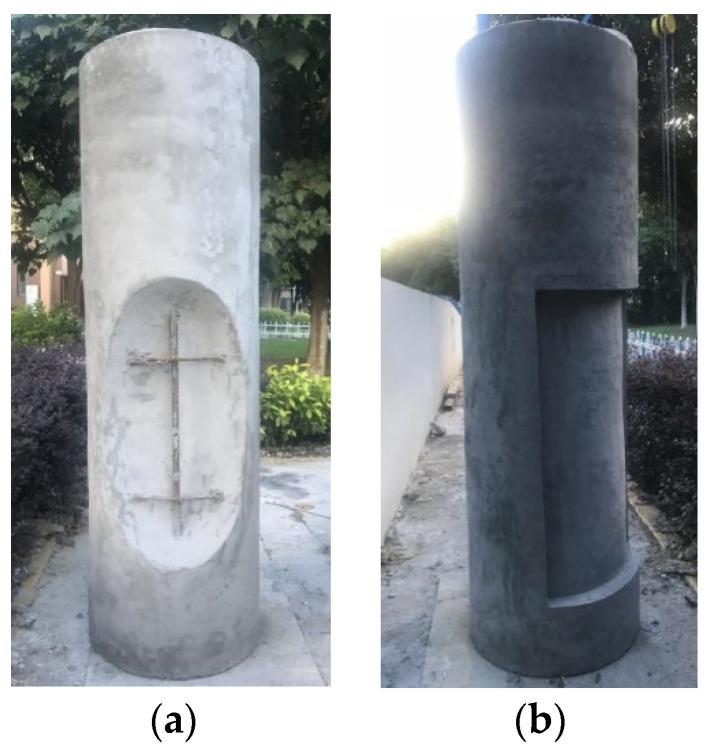
Concrete column with surface defects. (**a**) Hole. (**b**) Spalling.

**Figure 24:**
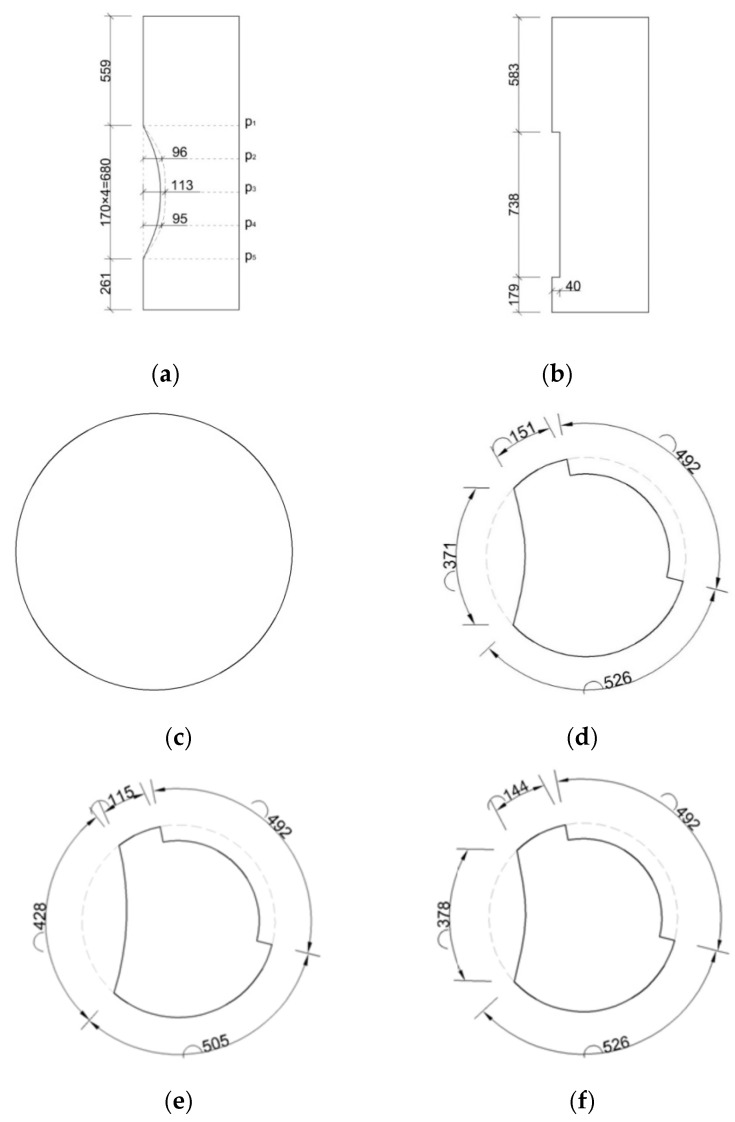

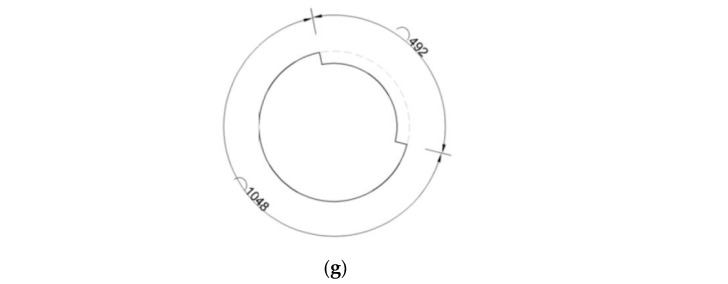
Surface defect dimensions (Unit: mm). (**a**) Hole dimensions. (**b**) Spalling dimensions. (**c**) Section p1. (**d**) Section p2. (**e**) Section p3. (**f**) Section p4. (**g**) Section p5.

Concrete column bridge pier models were designed in the laboratory, simulating two types of surface defects: holes and spalling. A concrete column bridge pier model was cast using C30 concrete with a diameter of 490 mm and a height of 1500 mm. The pier model was reinforced with longitudinal bars and hoop bars. The longitudinal bars consisted of 6 B16 steel bars, and the hoop bars consisted of 5 A8 steel bars. Additionally, the bridge pier model was designed to incorporate two types of surface defects: holes and spalling. Photos of the bridge pier model are shown in Figure 23, and detailed dimensions and the sizes of the surface defects are illustrated in Figure 24.

### 6.3. Layout of Measurement Points

The layout of measurement points involves determining the sonar scanning parameters, scanning distances, and the number and positions of measurement points, and calculating the azimuth and elevation of points.

(1) Azimuthal layout of measurement points

To obtain multi-point data for the underwater bridge pier model, the azimuthal layout of measurement points is calculated based on the requirements [4], known bridge pier model information, and the set sonar scanning parameters. Generally, the higher the sonar scanning frequency, the better the quality of the sonar image, but the scanning speed is relatively slower and the scanning range becomes smaller. To obtain clear sonar images, the scanning frequency is set to the maximum of 1.2 MHz, with a scanning speed of one sonar image every 5 s, which is acceptable. Due to the close scanning distance, the beamwidth at this frequency is 28° × 0.6°, which is also an acceptable scanning range. According to the previous model design, the radius of the pier is known to be r = 0.245 m.

Assuming that the horizontal beam angle’s edge line is tangent to the bridge piers surface, we can calculate *l_0_* as follows:

$$l_{0}=r\left(\frac{1}{\sin\left(\frac{\alpha_{0}}{2}\right)}-1\right)=0.245\times \left(\frac{1}{\sin\left(\frac{28{}^{\circ}}{2}\right)}-1\right)=0.768\;m$$

To obtain detailed apparent information from the pier’s surface, the distance *l* between the sonar measurement point and the pier’s surface is set to 0.5 m. Consequently, we can calculate *β* as follows:

$$\beta =2\arcsin\left[\left(1+\frac{l}{r}\right)\sin\left(\frac{\alpha_{0}}{2}\right)\right]-\alpha_{0}=66.7{}^{\circ}$$

Considering that the scanning range of the measurement points must completely cover the entire pier, and adding 2 more points for full coverage, we can determine the number of scanning points, *n*, as follows:

$$n=\left\lfloor \frac{360{}^{\circ}}{\beta }+0.5\right\rfloor +2=\left\lfloor \frac{360{}^{\circ}}{66.7{}^{\circ}}+0.5\right\rfloor +2=7$$

Here, ⌊ ⌋ represents the floor function. Of course, to increase the overlapping area, n can be further increased.

**Figure 25:**
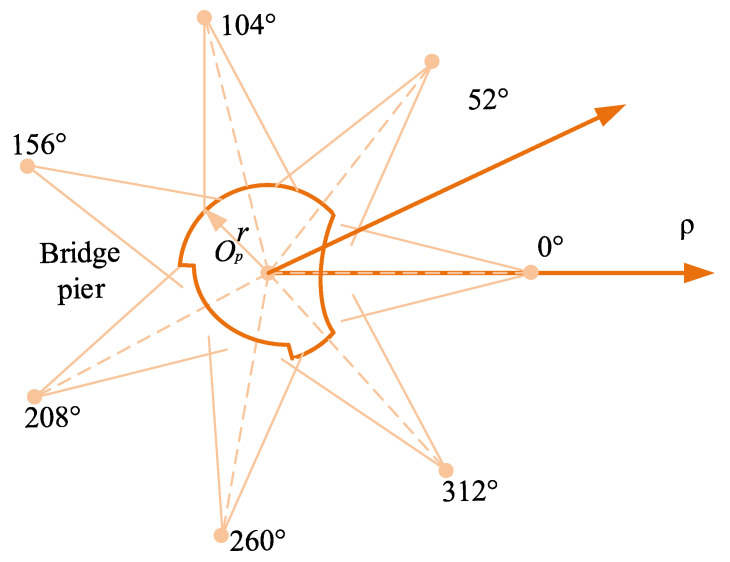
Azimuthal layout of measurement points.

Once *α*, *β*, *l*, and *n* are determined, the points are evenly distributed along the circular cross-section, with each point at the same distance from the pier’s edge. The angular distance between adjacent measurement points is equal, resulting in the azimuthal layout of measurement points for the bridge pier model, as shown in Figure 25.

(2) Vertical layout of measurement points

Given that the height of the bridge pier model is 1.088 m, the water depth is h = 1.400 m, and the sonar scanning radius is R = 1m, and considering the small size of the pier in the vertical direction, all measurement points are placed just 0.8 m below the water surface to ensure effective imaging.

### 6.4. Experimental Procedure

The experimental procedure involved several steps:

(1) Firstly, the turntable device was installed at the designated location. Next, the bridge pier model was suspended onto the turntable device using a hoisting bar. During the experiment, the turntable was rotated to change the position of the measurement points.

(2) Setting up the sonar system: Afterward, the sonar system was connected, and the sonar was mounted on the angle steel frame. The angle steel frame was securely fixed to the movable platform. Scanning of the bridge pier model was performed using horizontal and lateral scanning methods.

(3) Configuration of sonar parameters: The relevant parameters of the sonar system were configured using computer control software. The 1.2 MHz frequency, which provided better imaging results, was selected for scanning.

(4) Imaging scans: The imaging scans began at the 0° position, and the bridge pier model was scanned. After completing the scan at this point, the bridge pier model was rotated to 52°, and scanning at this measurement point commenced. This process continued, with imaging scans carried out at each measurement point, until all points were scanned.

**Figure 26:**
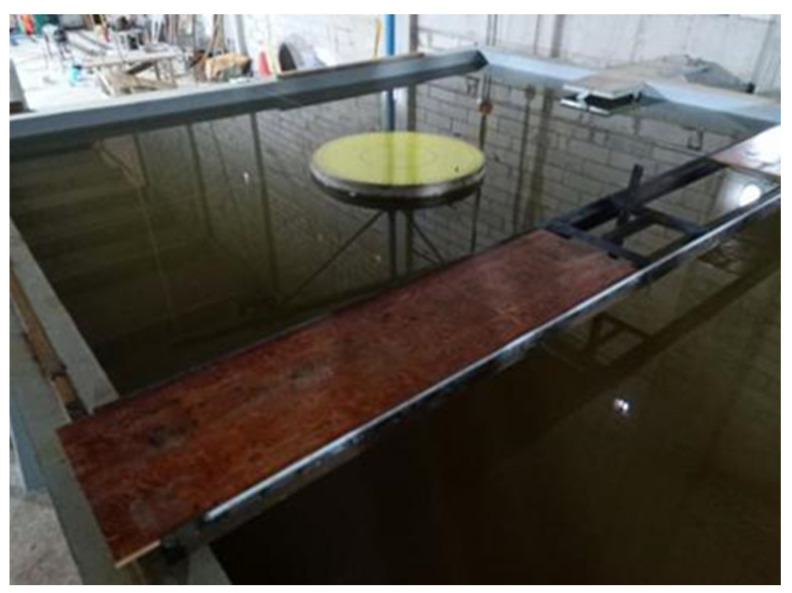
Test scene image.

The experimental setup and process are depicted in Figure 26 for reference.

### 6.5. Experimental Results

**Figure 27:**
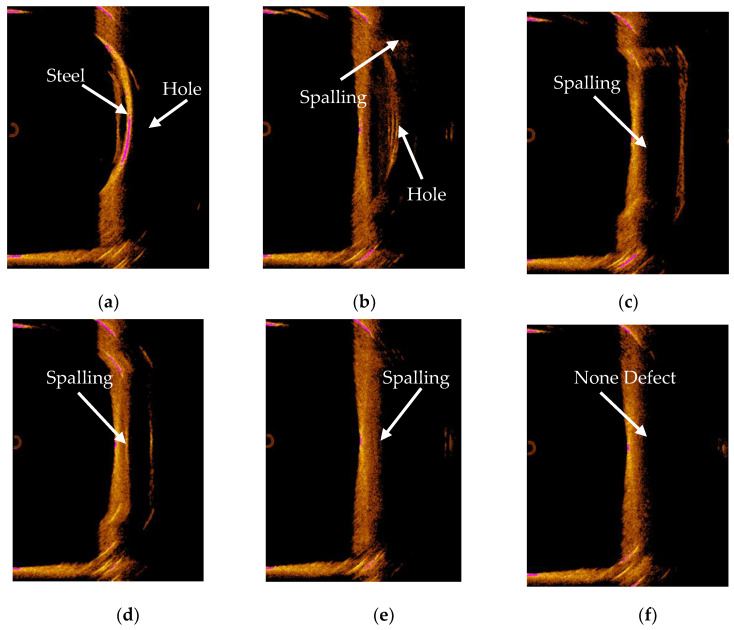

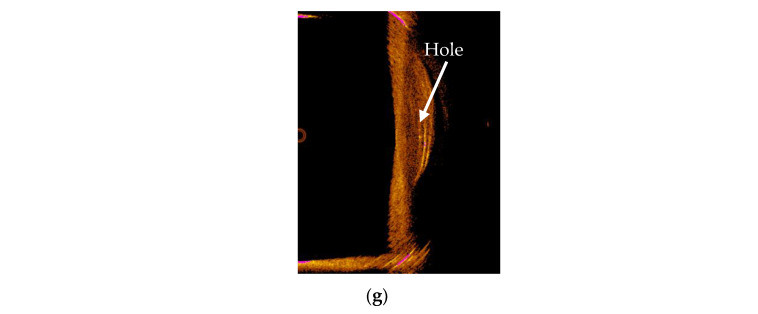
Sonar image of bridge pier model. (**a**) Sonar image of 0°. (**b**) Sonar image of 52°. (**c**) Sonar image of 104°. (**d**) Sonar image of 156°. (**e**) Sonar image of 208°. (**f**) Sonar image of 260°. (**g**) Sonar image of 312°.

The bridge pier model was subjected to imaging scans following the scanning plan, resulting in sonar images corresponding to various measurement points. These sonar images are shown in Figure 27.

When the bridge pier model at a measurement point did not exhibit any defects, the inner and outer contours of the bridge target in the sonar image appeared as straight lines, as seen in Figure 27f. This indicates that the bridge pier in that area has no surface defects.

However, when there were surface defects in the bridge pier model at a measurement point, both the inner and outer contours of the bridge target in the sonar image showed varying degrees of concavity and convexity, indicating the presence of surface defects in the bridge pier in that area.

Based on the sonar images, preliminary conclusions can be drawn as follows:

(1) Sonar images at 0°, 52°, and 312° correspond to areas with cavity surface defects.

(2) Sonar images at 52°, 104°, 156°, and 208° correspond to areas with spalling surface defects.

(3) The sonar image at 260° corresponds to an area with no surface defects.

These observations help to identify the presence or absence of surface defects in different regions of the bridge pier model.

**Figure 28:**
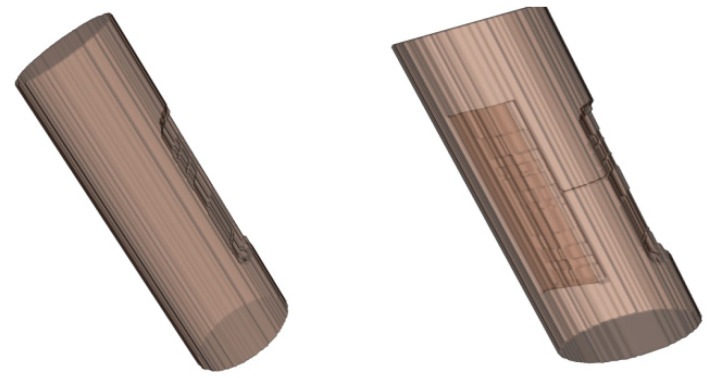
Reconstructed 3D model of the bridge pier.

By obtaining this set of sonar images, they can serve as input for the 3D reconstruction system. After undergoing image preprocessing in Section 3, sonar image 3D reconstruction in Section 4, and visualization in Section 5, the 3D model representing the surface defects in the underwater bridge pier has been reconstructed, as depicted in Figure 28.

### 6.6. Three-Dimensional Model Measurements

Using the generated underwater bridge pier 3D model, measurements of the surface defect portions were conducted to obtain positional information, 1D (distance measurement), 2D (area measurement), and 3D (volume measurement) data.

#### 6.6.1. Distance Measurement

As shown in Figure 28, the pixels on the reconstructed 3D model were converted into distances (with a conversion factor of 2.07 mm per pixel) and compared to the actual measurements from Figure 23, as presented in Table 2. It can be observed that there is a significant difference (15.04% difference) in identifying the exposed reinforcement as having no surface defects in the 0° sonar image. However, for the other geometric dimensions, the error does not exceed 12.00%. The reconstructed 3D model accurately represents the size of the actual bridge pier in most cases.

#### 6.6.2. Area Measurement

Using the scanned arc length of holes and spalls in the cross-section on the cylinder, the surface area can be calculated as follows:

(8) $$S=\sum_{i=1}^{x}l_{i}\cdot \Delta x$$

where S is the surface area. *l_i_* is the arc length on the cylinder when *x* = *i*. Δ*x* = 2.07 mm is the distance between two pixels in the x-direction.

A comparison of the calculated surface area of the model’s surface defects with the surface area obtained from actual measurements is shown in Table 3. The relative error for the hole area is −8.08%, and for the spalling area, it is −0.52%. The reconstructed model accurately reproduces the surface area of the bridge pier’s surface defects.

#### 6.6.3. Volume Measurement

Based on the surface area calculation Formula (8), we can calculate the volume using the following formula:

(9) $$V=\sum_{j=1}^{z}S_{j}\cdot \Delta z$$

where V is the volume. Δ*z* = 2.07 mm is the distance between two pixels in the z-direction. S*_j_* is the arc surface area when *z* = *j*.

A comparison of the calculated volume of the model’s defects with the volume obtained from actual direct measurements is shown in Table 4. The relative error for the hole volume is 13.56%, and for the spalling volume, it is 10.65%. The reconstructed model accurately reproduces the volume of the bridge pier’s defects. In practical engineering applications, when the defect size is small, it has a relatively minor impact on the pier’s load-bearing capacity and safety. When the defect size is large, despite the higher relative error, it still remains within acceptable limits. This is because, based on the load capacity experimental calculations, it is below the 15% safety assessment requirement [4]. This method has been successfully applied to actual engineering projects, including the Fuzhou Wulongjiang Bridge and the Fuqing Xizixiang Bridge sonar inspections, where the actual measurement results are consistent with the laboratory model test results.

The piers used in the experiment are scaled-down models of real bridge piers, and the accuracy of the 3D reconstruction algorithm depends on precise data. With sufficient accuracy, the research findings can be extrapolated beyond the confines of the specific experimental setup. The accuracy of sonar images depends on the frequency of the sonar; higher frequencies provide higher precision. In our experiment, we selected the highest frequency available for this particular sonar model.

## 7. Conclusions

This study has summarized the research on the 3D digital reconstruction and visualization of sonar images depicting surface defects on underwater bridge piers. The following key conclusions have been drawn from this research:

(1) We have introduced an automated method for recognizing the surface contours of underwater bridge piers. This method incorporates a series of steps in sonar image processing, including histogram equalization, wavelet threshold denoising, image segmentation, binarization, hole filling, mathematical morphology operations, and contour extraction. Importantly, this automated recognition method has been seamlessly integrated into digital inspection software, facilitating the accurate and automated extraction of underwater bridge pier surface contours.

(2) The study has presented a 3D reconstruction algorithm tailored for underwater bridge piers’ surface defects. Leveraging contour information extracted from sonar images as control points and combining it with existing non-destructive bridge pier model data, this algorithm effectively reconstructs various 3D models representing different types of surface defects.

(3) We have developed 3D visualization software for underwater bridge piers based on sonar images, utilizing the Visualization Toolkit (VTK). This software adopts surface rendering techniques and leverages VTK’s visualization capabilities to display 3D models of underwater bridge piers. Furthermore, it offers interactive functionalities such as translation, scaling, and rotation, enhancing the convenience of researchers in analyzing and observing surface defects within underwater bridge pier 3D models.

(4) The effectiveness of the proposed 3D model reconstruction technology has been successfully validated through experiments. The experimental results unequivocally demonstrate the capacity of the reconstructed models to faithfully represent the 3D surface defects on underwater bridge piers. Importantly, the relative errors in surface defect measurements remain within acceptable limits, affirming the reliability and efficacy of this technology.

In conclusion, this research has furnished robust tools and methodologies for digitally detecting and managing surface defects in underwater bridge piers. The practical engineering applications of this research hold substantial promise for the field. In conclusion, this study has equipped us with robust tools and methodologies for digitally detecting and managing surface defects in underwater bridge piers. The practical engineering applications of this research show substantial promise for the field. Nonetheless, there remain unresolved issues, such as integrating optical and acoustic detection technologies to develop integrated opto-acoustic devices capable of operating effectively in complex underwater environments, thereby accurately locating and identifying surface defects on underwater bridge piers. The 2D sonar image-based 3D reconstruction method proposed in this paper relies on recognizing bridge pier cross-sectional profiles. It was initially designed to accurately reconstruct various types of basic defects with minimal image data. However, compared to 3D reconstruction methods using 3D sonar, the resulting 3D models are still relatively coarse.

## Figures and Tables

**Table 1:** Evaluation metrics of different denoising methods.

Denoising Methods	MSE	PSNR	SNR
Noisy Sonar Images	0.0071	69.6331	1.8188
Basic Mean Filtering	0.0042	72.968	5.1538
Basic Median Filtering	0.002	75.138	7.3237
Hard Thresholding Function	0.0032	74.968	6.1538
Soft Thresholding Function	0.0018	76.7792	8.9649
Garrote Function	0.0036	73.5295	5.7152
Proposed Algorithm	0.0017	76.9467	9.0899

**Table 2:** Distance measurement results.

	Actual Distance A (mm)	3D Model Distance B (mm)	Relative Error (%) (1 − A/B) × 100
Hole maximum height	680	682	0.29
Hole maximum arc height	428	395	−8.35
Hole maximum depth	113	133	15.04
Spalling maximum height	738	751	1.73
Spalling maximum arc height	680	682	0.29
Spalling maximum depth	428	395	−8.35

**Table 3:** Comparison of defects surface area between reconstructed model and actual pier.

	Actual Pier Surface Area S_a_(m^2^)	3D model Surface Area S_b_/(m^2^)	Relative Error (%) (1 − S_a_/S_b_) × 100
Hole	0.20009	0.18513	−8.08
Spalling	0.36310	0.36123	−0.52

**Table 4:** Comparison of defects volumes between the reconstructed model and the actual pier.

	Actual Pier Volumes V_cal_ (m^3^)	3D Model Volumes V_meas_ (m^3^)	Relative Error (%) (1 − V_cal_/V_meas_) × 100
Hole	0.20009	0.18513	−8.08
Spalling	0.36310	0.36123	−0.52

## Data Availability

The data are available from the National Natural Science Foundation of China (No. 52278295); the Natural Science Foundation of Fujian Province, China (No. 2022J02016); the Talent Start-up Fund Project of Fuzhou University (No. XRC-23030).
